# Administration of *N*-Acyl-Phosphatidylethanolamine Expressing Bacteria to Low Density Lipoprotein Receptor^−/−^ Mice Improves Indices of Cardiometabolic Disease

**DOI:** 10.1038/s41598-018-37373-1

**Published:** 2019-01-23

**Authors:** Linda S. May-Zhang, Zhongyi Chen, Noura S. Dosoky, Patricia G. Yancey, Kelli L. Boyd, Alyssa H. Hasty, MacRae F. Linton, Sean S. Davies

**Affiliations:** 1 Division of Clinical Pharmacology, Department of Pharmacology, 2220 Pierce Avenue, Vanderbilt University, 556 Robinson Research Building, Nashville, TN 37221 USA; 2 Department of Medicine, Division of Cardiovascular Medicine, Vanderbilt Medical Center, 2220 Pierce Avenue, 312 Preston Research Building, Nashville, TN 37232 USA; 3 AA-6206 Medical Center North, Department of Pathology, Microbiology, and Immunology, Vanderbilt Medical Center, 1211 Medical Center Drive, Nashville, TN 37232 USA; 4 Department of Molecular Physiology and Biophysics, Vanderbilt University, 2220 Pierce Avenue, 813 Light Hall, Nashville, TN 37232 USA

## Abstract

Obesity increases the risk for cardiometabolic diseases. *N*-acyl phosphatidylethanolamines (NAPEs) are precursors of *N*-acylethanolamides, which are endogenous lipid satiety factors. Incorporating engineered bacteria expressing NAPEs into the gut microbiota retards development of diet induced obesity in wild-type mice. Because NAPEs can also exert anti-inflammatory effects, we hypothesized that administering NAPE-expressing bacteria to low-density lipoprotein receptor (*Ldlr*)^−/−^ mice fed a Western diet would improve various indices of cardiometabolic disease manifested by these mice. NAPE-expressing *E. coli Nissle 1917* (*pNAPE-EcN*), control *Nissle 1917* (*pEcN*), or vehicle (veh) were given via drinking water to *Ldlr*^−/−^ mice for 12 weeks. Compared to *pEcN* or *veh* treatment, *pNAPE-EcN* significantly reduced body weight and adiposity, hepatic triglycerides, fatty acid synthesis genes, and increased expression of fatty acid oxidation genes. *pNAPE-EcN* also significantly reduced markers for hepatic inflammation *a*nd early signs of fibrotic development. Serum cholesterol was reduced with *pNAPE-EcN*, but atherosclerotic lesion size showed only a non-significant trend for reduction. However, *pNAPE-EcN* treatment reduced lesion necrosis by 69% indicating an effect on preventing macrophage inflammatory death. Our results suggest that incorporation of NAPE expressing bacteria into the gut microbiota can potentially serve as an adjuvant therapy to retard development of cardiometabolic disease.

## Introduction

Obesity frequently associates with adverse physiological changes including hypertension, hypertriglyceridemia, hypercholesterolemia, and insulin resistance. These changes, commonly termed metabolic syndrome, increase the risks for atherosclerosis, type 2 diabetes, and non-alcoholic fatty liver disease (NAFLD). The rapid rise in obesity in the Western world threatens the decline in mortality from atherosclerosis, the leading cause of death worldwide^1^, which have occurred in the past two decades due to cholesterol-lowering drugs such as statins^2^. Dysregulated lipid metabolism accompanied by chronic inflammation is central to the development of atherosclerotic plaques. Subsequent formation of necrotic cores and rupture of these vulnerable atherosclerotic plaques are thought to be critical steps leading to thrombosis, myocardial infarction, and death. The rise in obesity has also markedly increased the prevalence of type 2 diabetes and led to NAFLD becoming the most common cause of abnormal liver function, with 38% of adults in the United States affected^3^. While early stages of NAFLD are considered relatively benign by clinicians, progression to chronic liver inflammation (non-alcoholic steatohepatitis, NASH), fibrosis, and cirrhosis significantly impacts functionality and lifespan.

One novel therapeutic target for slowing development of metabolic syndrome and cardiometabolic disease is the gut microbiota, as the composition and functionality of the gut microbiota differs in individuals with obesity^4–7^, atherosclerosis^8^, type 2 diabetes^9,10^, and/or NAFLD^11^ compared to their healthy counterparts. Because the gut microbiota chronically releases metabolites that affect various host cells, small but sustained changes in bacterial metabolites can significantly impact disease progression. Recently, we engineered a commensal *E. coli* strain (*Nissle 1917*) to produce *N*-phosphatidylethanolamines (NAPEs), and demonstrated that incorporating these therapeutically modified bacteria (*pNAPE-EcN*) into the gut microbiota retarded the development of obesity and glucose intolerance in wildtype mice fed a high fat diet^12^. NAPEs are endogenous anorexigenic lipids normally synthesized by enterocytes of the small intestine (primarily in the duodenum and jejunum) in response to feeding^13–15^. However, a diet chronically high in fat impairs this intestinal biosynthesis of NAPEs^16–18^. The mechanisms underlying this impaired biosynthesis are unknown. We reasoned that bacterially-synthesized NAPEs could be used to compensate for this reduced NAPE synthesis. Interestingly, incorporation of NAPE expressing bacteria into the microbiota of the gut (primarily in the large intestine) proved more potent and efficacious than oral administration of purified NAPE^19^.

While administration of NAPEs is sufficient to exert therapeutic effects^16^, NAPEs undergo hydrolysis to *N*-acyl-ethanolamides (NAEs) by NAPE-hydrolyzing phospholipase D (NAPE-PLD). We found that the anti-obesity effects of our engineered bacteria require NAPE-PLD^19^, which supports the notion that NAPEs serve as precursors for NAEs rather than exerting effects directly. NAEs such as C18:1NAE (*N*-oleoylethanolamide) are ligands for receptors including PPARα, GPR119, and TRPV1. C18:1NAE reduces food intake and increases in fatty acid oxidation, leading to reduced serum triglycerides, and these effects are blunted in PPARα^−/−^ mice^20^. Activation of PPARα by various agonists induces ApoAI expression^21^ and increase HDL levels *in vivo*^22^. C18:1NAE also stimulates GLP-1 release via GPR119 and activation of GPR119 promotes glucose-stimulated insulin secretion^23^. GPR119 activation also promotes ABCA1 expression in macrophages and therefore promotes ABCA1-mediated cholesterol efflux by HDL from macrophages^24^. NAEs also exert direct anti-inflammatory effects^25–35^. Based on these various effects, increasing NAPE and NAE levels would be anticipated to protect against metabolic syndrome and cardiovascular disease, and in fact, intraperitoneal injection of NAEs in mice can reduce steatosis^36^, lower LDL levels^37^, and reduce atherosclerosis^38,39^.

We hypothesized that administration of NAPE expressing bacteria might be a sustainable, long-term intervention to retard the progression of metabolic syndrome and cardiometabolic disease. To test this hypothesis, we used low density lipoprotein receptor null mice (*Ldlr*^−/−^), as these mice manifest a number of key aspects of cardiometabolic disease when fed the Western diet. For instance, while feeding a Western diet to wild-type mice induces adiposity, glucose intolerance, and hepatosteatosis, it fails to initiate significant hepatic inflammation or atherosclerosis^40^. In contrast, feeding the Western diet (but not a low fat diet) to *Ldlr*^−/−^ mice initiates steatohepatitis (NASH)^41–43^ and atherosclerosis^38,44,45^. We examined the effects of administering *pNAPE-EcN* on the development of various indices of cardiometabolic disease in these *Ldlr*^−/−^ mice, and found significant improvements not only in adiposity, but also in reduced hepatic triglycerides, reduced hepatic inflammation and macrophage infiltration, and reduced necrosis of atherosclerotic lesions.

## Results

### pNAPE-EcN reduces body weight and adiposity gain independent of food intake

**Figure 1:**
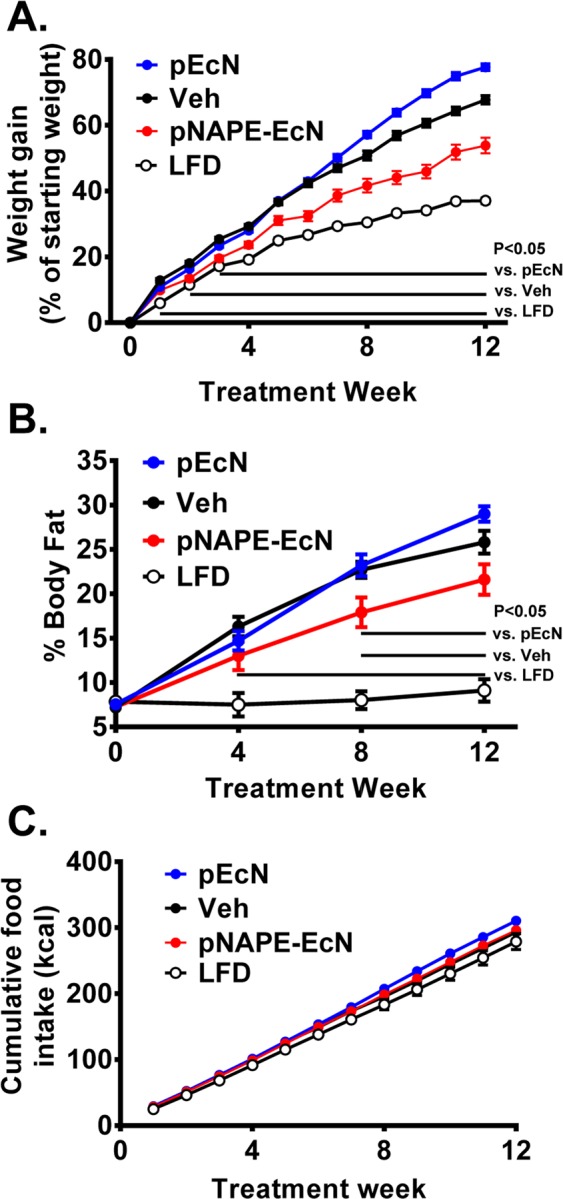
*pNAPE-EcN*, but not *pEcN*, inhibits gain in body weight and adiposity, independent of food intake. All values are mean ± SEM (n = 10 mice per group). *pEcN*, Veh, and *pNAPE-EcN* groups were fed WD for 12 weeks and compared to LFD as an additional control group. (**A**) Effect on % gain of body weight from start of treatment. (**B**) Effect on % body fat. (**C**) Effects on cumulative food intake by energy. Solid bars indicate time points with significant differences (P < 0.05) between *pNAPE-EcN* and other groups (2-way repeated measures ANOVA with Dunnett’s multiple comparison test). In addition to these differences relative to *pNAPE-EcN*, *pEcN* differed vs Veh P < 0.05 starting at 8 weeks for % gain of body weight. LFD differed vs all WD groups P < 0.05 starting at 1 week for % gain of body weight and at 4 weeks for % body fat.

Previously, we showed that administration of a probiotic strain of *E. coli*, *Nissle 1917* (*EcN*) transformed with a plasmid for expression of *A. thaliana NAPE synthase* Atlg78690 (*pNAPE-EcN*) in drinking water inhibited development of obesity in C57BL/6J wildtype mice fed a high-fat diet^12^. Similar to C57BL6 mice, *Ldlr*^−/−^ mice treated with *pNAPE-EcN* gained relatively less body weight (Fig. 1A) (versus *Veh* 2 weeks, P < 0.05; versus *pEcN* 3.5 weeks, P < 0.05) and accumulated relatively less body fat (Fig. 1B) (versus *Veh* 4 weeks, P < 0.05; versus *pEcN* 8 weeks, P < 0.05) compared to vehicle treated mice fed the Western diet during the 12 week treatment period. *pNAPE-EcN* treatment had no effect on food intake (Fig. 1C). Raw values for change in body weight and fat mass are depicted in Supplementary Fig. 1. Furthermore, *pNAPE-EcN* treated animals had lower fasting blood glucose levels than *Veh* treated (153.6 ± 6.9 vs 189.7 ± 6.4 mg/ml, P < 0.05) at levels similar to mice fed *LFD* (149.7 ± 7.0 mg/dl) at 8 weeks (Supplementary Fig. 2).

### pNAPE-EcN increases hepatic and adipose NAEs

**Figure 2:**
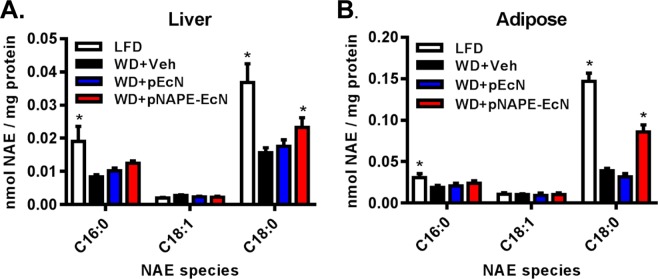
*pNAPE-EcN* increased hepatic and adipose NAE levels at the end of the 12-week study. Values are represented as mean ± SEM. Statistical significance is *P < 0.05 by 2-way ANOVA with Dunnett’s multiple comparisons test and denotes comparing to Western diet + *Veh*.

Bacterial NAPEs absorbed by the intestinal tract are converted into NAEs by NAPE-PLD, resulting in increased levels in liver and adipose tissue^12,19^. The most prominent NAE species detected in liver of all groups was C18:0NAE and mice fed the Western diet had markedly reduced hepatic NAE levels compared to those fed LFD (Fig. 2). Treatment with *pNAPE-EcN* significantly increased (P < 0.05) C18:0NAE levels compared to vehicle treated mice, although these levels were still less than the LFD fed group. The Western diet also markedly reduced NAE levels in adipose tissue compared to LFD, and again treatment with *pNAPE-EcN* treatment increased C18:0NAE levels compared to vehicle treated mice (Fig. 2). Taken together these data suggest that the Western diet markedly reduces endogenous NAE biosynthesis, which is consistent with previous studies in wild-type mice using high fat diets^16–18^, and that *pNAPE-EcN* treatment partially compensates for this loss.

### pNAPE-EcN inhibits the accumulation of liver TG and reduces hepatic inflammation and fibrosis

**Figure 3:**
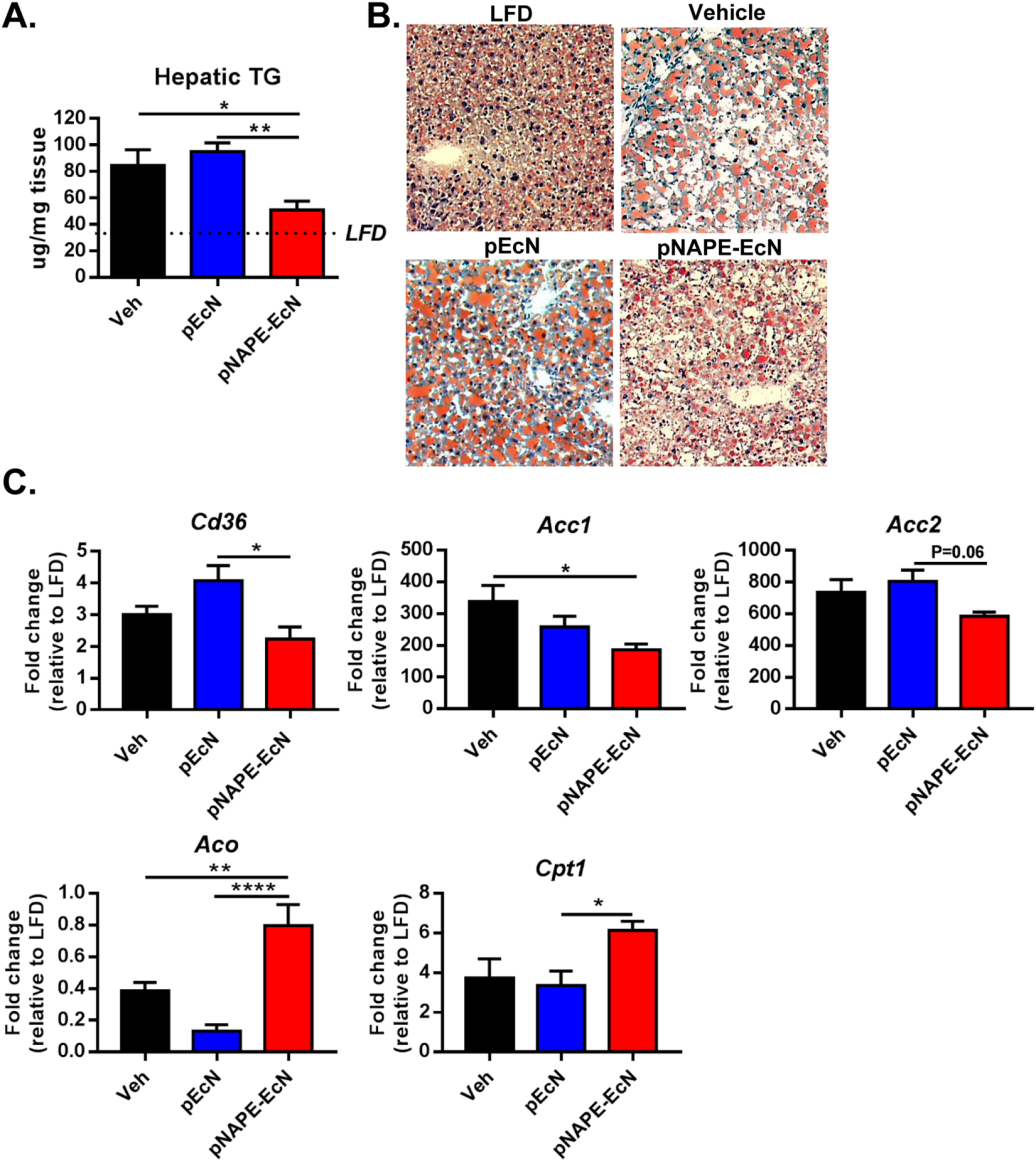
*pNAPE-EcN* reduces development of diet-induced hepatosteatosis. (**A**) Effect of treatments (n = 9–10 animals per group) on hepatic TG levels at the end of the 12-week study. (**B**) Representative images of liver sections stained with Oil Red O. (**C**) Effect of treatments on hepatic mRNA expression of *Cd36*, *Acc1*, *Acc2*, *Aco*, and *Cpt1a*. Values are represented as mean ± SEM. Dotted line represents animals on low fat diet as a comparison. Statistical significance is *P < 0.05; **P < 0.01; ****P < 0.001 by 1-way ANOVA with Dunnett’s multiple comparisons test.

In contrast to wildtype mice, *Ldlr*^−/−^ mice on a Western diet manifest hepatic inflammation and early fibrosis that mark the progression from simple steatosis towards NASH. We therefore sought to determine the effect of *pNAPE-EcN* on this progression. Hepatosteatosis manifests as a highly vacuolated liver. Animals fed a Western diet and treated with *vehicle* and *pEcN* displayed multiple hallmarks of hepatosteatosis including markedly elevated hepatic TG levels (Fig. 3A) and highly vacuolated morphology with lipid accumulation (Fig. 3B) compared to animals fed LFD. In contrast, mice treated with *pNAPE-EcN* showed marked reductions in hepatic TG levels (Fig. 3A) (P < 0.05 vs. *Veh*; P < 0.01 vs. *pEcN*), improved hepatic morphology, and less lipid accumulation (Fig. 3B). *pNAPE-EcN* treatment reduced hepatic expression of fatty acid transporter *Cd36* (versus *pEcN*, P < 0.05) as well as fatty acid synthesis gene *acetyl-CoA carboxylase 1* (*Acc1*) (versus *Veh*, P < 0.05) and tended to reduce *Acc2* (versus *pEcN*, P = 0.06) (Fig. 3C). *pNAPE-EcN* treatment increased the hepatic expression of genes involved in fatty acid oxidation, *acyl-coA oxidase* (*Aco*) (versus *Veh*, P < 0.01; versus *pEcN*, P < 0.001) and *carnitine acyltransferase 1a* (*Cpt1a*) (versus *pEcN*, P < 0.01) (Fig. 3C). Hepatic expression of *peroxisome proliferator-activated receptors* (*Pparα, Pparδ, Pparγ)* and *lipoprotein lipase (Lpl)* were not different among any of the groups fed the Western diet (Supplementary Table 2).

**Figure 4:**
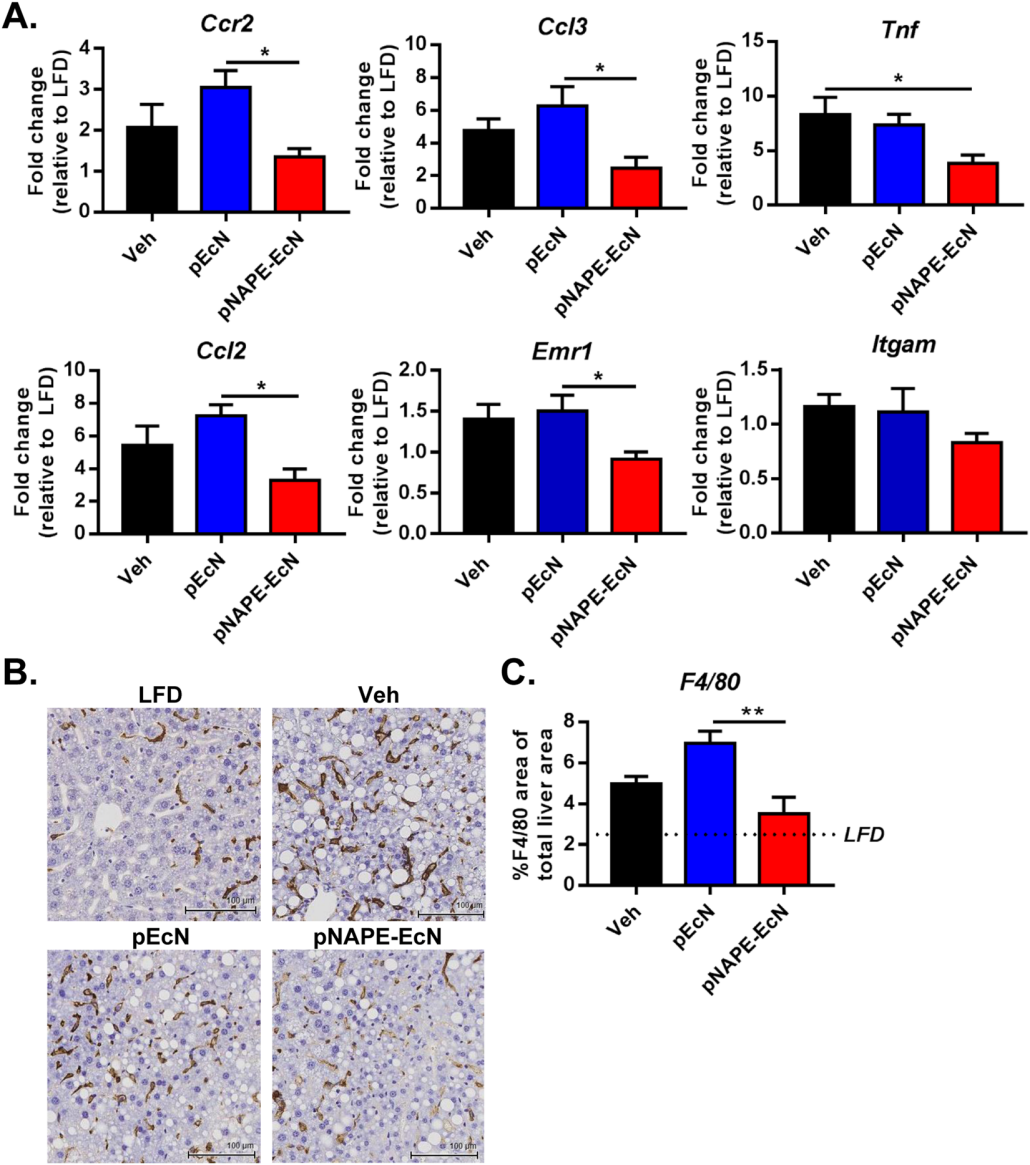
*pNAPE-EcN* reduces diet-induced hepatic inflammation. (**A**) Effect of treatments on expression of hepatic genes involved in inflammation and macrophage infiltration at the end of the 12-week study. Values are represented as mean ± SEM. Dotted lines represent animals on a low fat diet. Statistical significance is *P < 0.05 by 1-way ANOVA with Dunnett’s multiple comparisons test. (**B**) Representative images of liver sections immunostained for F4/80. (**C**) Digital quantitation of F4/80-stained slides expressed as % F4/80-stained area of total liver area.

*pNAPE-EcN* treatment reduced molecular markers of diet-induced hepatic inflammation, as indicated in reduced hepatic mRNA expression for genes linked to monocyte and macrophage infiltration (Fig. 4A) such as *C-C motif ligand 2* (*Ccl2*, also known as *monocyte chemoattractant protein 1* or *Mcp1*), *C-C chemokine receptor type 2* (*Ccr2*, the receptor for CCL2), and *C-C motif ligand 3* (*Ccl3*, also known as *macrophage inflammatory protein 1-alpha* or *Mip1a*) compared to animals treated with *pEcN*. The expression of both *tumor necrosis factor* (*TNFα*), and *EGF-like module-containing mucin-like hormone receptor-like 1* (*Emr1*, also known as *F4/80*) was significantly reduced with *pNAPE-EcN* treatment, while the expression of *Integrin subunit alpha M* (*Itgam*, also known as *Cd11b*) only tended to be reduced. Immunostaining for infiltrating monocytes/macrophages using antibodies against *F4/80* revealed that the livers of control mice have large numbers of immunoreactive cells, while *pNAPE-EcN* treated mice had markedly fewer immunoreactive cells (versus *pEcN*, P < 0.01) (Fig. 4B,C). Hepatic expression of *Il-6* and *Cd68* were unchanged among any of the groups fed the Western diet (Supplemental Table 2). *pEcN* increased *Il-10* expression compared to both vehicle-treated (P < 0.001) and *pNAPE-EcN*-treated groups (P < 0.001) (Supplemental Table 2). Taken together, our data show that *pNAPE-EcN* markedly reduces diet-induced hepatic inflammation.

**Figure 5:**
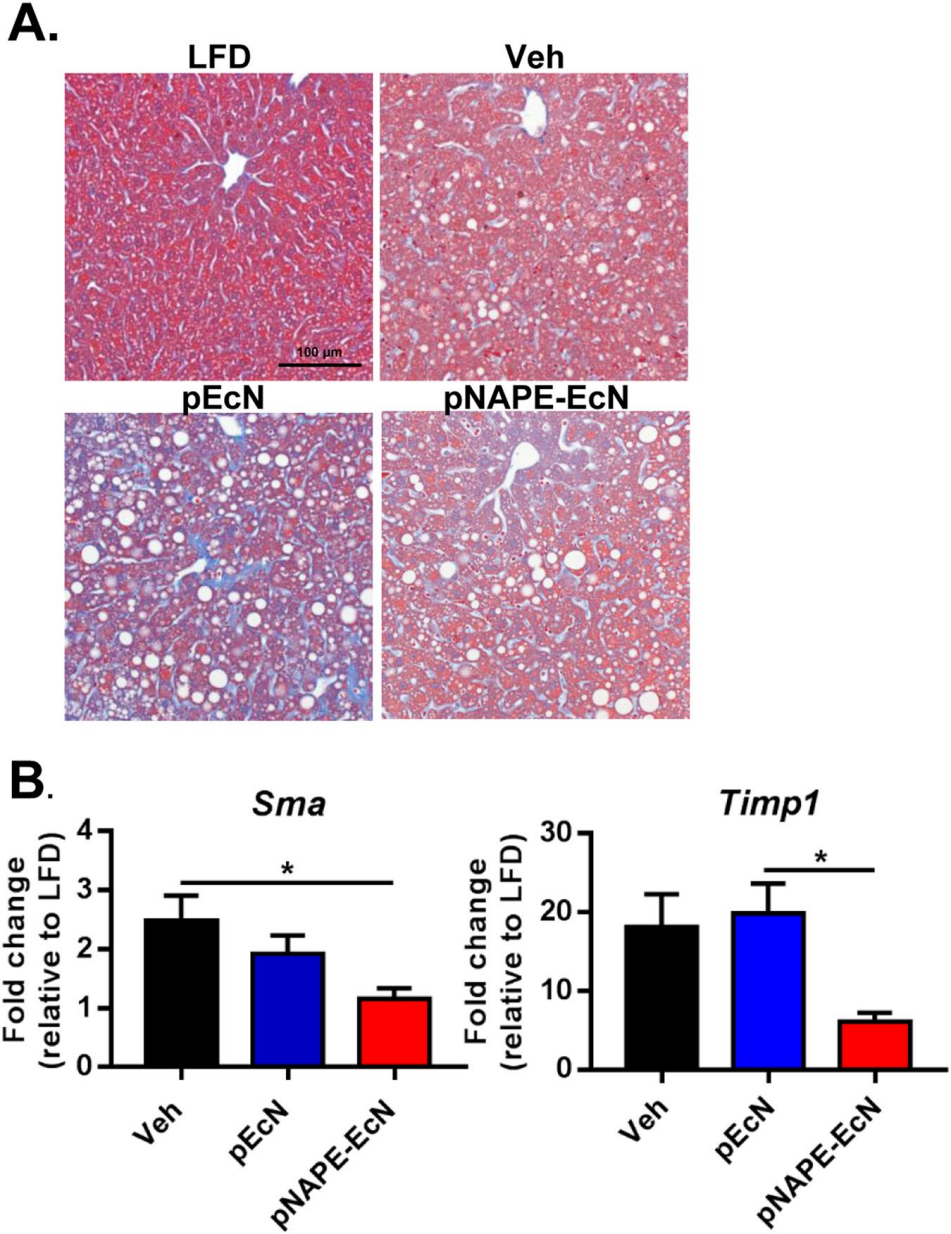
*pNAPE-EcN* reduces initiation of hepatic fibrosis. (**A**) Representative images of Trichrome Blue staining of liver sections. (**B**) Effects of treatment on hepatic gene expression of smooth muscle actin and tissue inhibitor of metalloproteinase 1 at the end of the 12-week study. Values are represented as mean ± SEM. Dotted lines represent animals on a low fat diet. Statistical significance is *P < 0.05 by 1-way ANOVA with Dunnett’s multiple comparisons test.

Cytochemical staining with Trichrome Blue revealed only modest fibrosis even in the control *Ldlr*^−/−^ mice fed the Western diet (Fig. 5A), with some mild fibrosis observed around vessels and in sinusoids of *pEcN* treated mice. Despite this lack of visually observable fibrosis, molecular markers indicating the initiation of fibrosis were elevated in vehicle treated mice and suppressed in *pNAPE-EcN* treated mice. For instance, increased mRNA levels of *alpha-smooth muscle actin* (*Sma*)^46,47^ and *tissue inhibitor of metalloproteinases-1* (*Timp1*)^48^ are both early indicators for progression towards hepatic fibrosis. Hepatic expression of *Sma* increased in mice fed a Western diet and treated with either vehicle or control (P < 0.05), while mice treated with *pNAPE-EcN* had *Sma* levels similar to the LFD group (Fig. 5B). Mice treated with *pNAPE-EcN* also had reduced hepatic gene expression of *Timp1* (Fig. 5B). Hepatic expression of *transforming growth factor β* (*Tgfβ)* was unchanged among groups (Supplemental Table 2). Taken together, the data suggest that *pNAPE-EcN* treatment slowed initiation of hepatic fibrosis.

### pNAPE-EcN reduces atherosclerotic lesion necrosis

**Figure 6:**
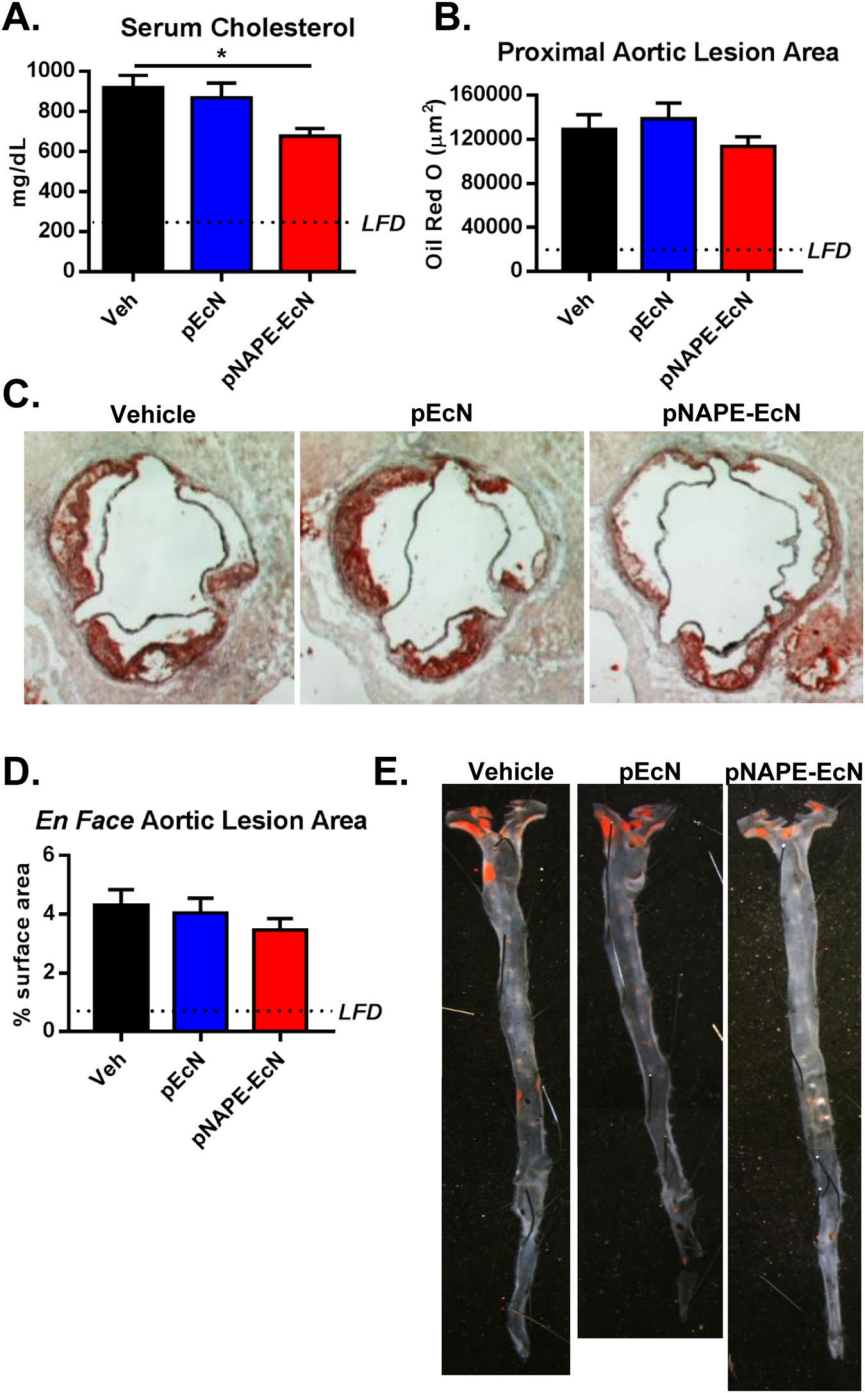
Effects of *pNAPE-EcN* on serum cholesterol levels and atherosclerotic lesion size at the end of the 12-week study. (**A**) Serum total cholesterol levels were determined by enzymatic methods. Absorbance was measured at 500 nm. (**B**) Effect of treatments on proximal aortic lesion area as determined by Oil Red O staining. (**C**) Representative images of Oil Red O stained proximal aortic sections. (**D**) Effect of treatments on atherosclerosis determined by en face analysis of Sudan IV stained aortas. (**E**) Representative images of Sudan IV stained aortas. Dotted lines represent animals on a low fat diet. Values are represented as mean ± SEM. *P < 0.05. Statistical significance by 1-way ANOVA with Dunnett’s multiple comparisons test.

**Figure 7:**
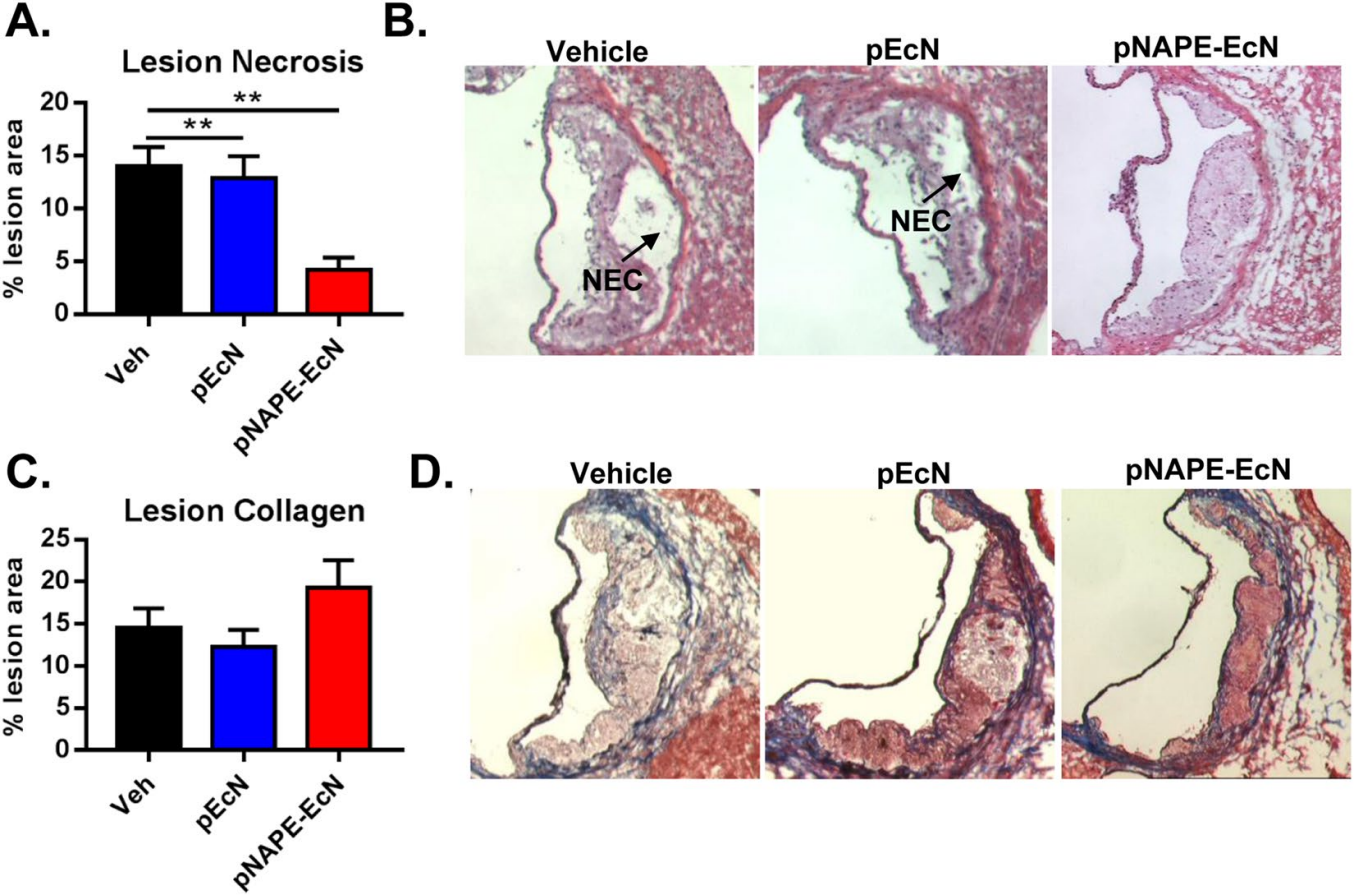
Effects of *pNAPE-EcN* on lesion necrosis and collagen at the end of the 12-week study. (**A**) Effect of treatments (n = 8 animals per group) on cross-sectional necrotic area (NEC) determined by a lack of H&E staining in the lesion area. (**B**) Representative images of H&E stained slides. (**C**) Effect of treatments on lesion collagen content determined by Trichrome Blue. (**D**) Representative images of Trichrome blue staining. Values are represented as mean ± SEM.

In humans, lack of functional LDLR markedly increases plasma cholesterol and atherosclerosis, leading to necrotic plaques vulnerable to rupture and which causes thrombosis, vessel occlusion, and myocardial infarctions. *Ldlr*^−/−^ mice on a Western diet have markedly elevated plasma cholesterol levels and their atherosclerotic plaques progress to the early stages of necrosis. We determined the effect of *pNAPE-EcN* treatment on atherosclerotic lesion size and progression in these mice. Treatment with *pNAPE-EcN* reduced plasma cholesterol levels of mice fed a Western diet, compared with those treated with vehicle or *pEcN* (Fig. 6A). Oil Red O staining of proximal aortic sections showed a non-significant trend for a reduction in atherosclerotic lesion size for *pNAPE-EcN* treated mice compared to *vehicle* or *pEcN* (Fig. 6B,C). Similarly, *pNAPE-EcN* caused a non-significant trend for a reduction in atherosclerotic lesion area as examined by *en face* analysis of Sudan IV stained aortas (Fig. 6D,E). Despite the lack of significant changes in aortic lesion size, *pNAPE-EcN* treatment markedly decreased the necrotic area within the atherosclerotic lesion quantified by H&E staining by 69% (Fig. 7A,B) (P < 0.001). Lesional collagen content tended to be increased with *pNAPE-EcN* treatment compared to *vehicle* or *pEcN* but the difference was not statistically significant (Fig. 7C,D). These results suggest that while the modest reduction in cholesterol levels induced by *pNAPE-EcN* did not significantly impact atherosclerotic lesion development, the anti-inflammatory effects of NAEs prevented the formation of more vulnerable necrotic plaques.

## Discussion

*Ldlr*^−/−^ mice fed a Western diet manifest a number of key features of human cardiometabolic disease and show a marked diminution in tissue NAE levels, so they represent an important model for determining the potential of NAPE expressing bacteria to treat human disease. Our finding that feeding the Western diet reduced liver and adipose NAE levels in these mice is consistent with previous studies showing that high fat diets reduced NAPE and NAE levels, at least in the small intestine^16–18^. The mechanisms of this diet induced reduction in biosynthesis is not known and warrants further study. However, that this reduction may have important implications for cardiometabolic disease is shown by our findings that treatment of *Ldlr*^−/−^ mice with *pNAPE-EcN* was able to partially restore tissue NAE levels and markedly reduced a number of key markers of cardiometabolic disease in these mice, such as hepatosteatosis, inflammation, and the formation of necrotic atherosclerotic plaques. Thus, incorporating NAPE expressing bacteria into the gut microbiota has potential as an adjuvant therapy to slow the progression of cardiometabolic disease. These studies extend our previous findings showing that incorporating NAPE expressing bacteria into the gut microbiota slowed the development of obesity, glucose intolerance, and insulin resistance in wildtype C57BL/6 mouse fed a high fat diet.

Although previous studies indicated that administration of NA(P)Es protect against obesity and hepatosteatosis^12,36^, our findings with *Ldlr*^−/−^ mice provide additional insights into the mechanisms whereby NA(P)Es exert protective effects in the liver. *pNAPE-EcN* did not alter food intake in the *Ldlr*^−/−^ mice, in contrast to wild-type mice fed a high-fat diet where reduced food intake significantly contributed to the reduced hepatosteatosis induced by *pNAPE-EcN*^12^. The mechanism underlying the loss of this anorexic effect of *pNAPE-EcN* in *Ldlr*^−/−^ mice is not clear, but previous studies have demonstrated that activation of PPARα induces *Ldlr* expression^49^. Since the loss of *Ldlr* has previously been shown to alter feeding behavior and sensitivity to leptin under some conditions^50^, we speculate that the NAPE/NAE/PPARα pathways leading to reduced food intake in wild-type mice is dependent in some way on functional LDLR. Future studies are needed to elucidate this relationship.

In the *Ldlr*^−/−^ mice, clearly other mechanisms besides a reduction in food intake must drive the reduced body weight gain and hepatic fat accumulation. Although we did not measure energy expenditure and resting metabolic rate in these experiments, our previous studies suggest that these could be key mechanisms. In wild-type mice, treatment with *pNAPE-EcN* increased resting metabolic rate (but not physical activity) in addition to reducing food intake, and increased expression of fatty acid oxidation genes such as *Aco* seemed to underlie the increased resting metabolic rate. In the *Ldlr*^−/−^, mice, treatment with *pNAPE-EcN* also increased expression of *Aco and Cpt1a*, as well as decreased hepatic expression of *Cd36, Acc1, and Acc2*. Therefore, we speculate that *Ldlr*^−/−^ mice treated with *pNAPE-EcN* may also have increased resting metabolic rates which would then account for their reduced adiposity despite no change in food intake.

Previous studies suggest that such changes can protect against hepatosteatosis. CD36 serves as a transporter of long chain fatty acids and as a receptor for these ligands that trigger phospholipase C activity and calcium signaling^51^. Liver specific deletion of *Cd36* in mice fed a high fat diet markedly reduces hepatic accumulation of TG and expression of inflammatory markers^52^. In human patients undergoing bariatric surgery for morbid obesity, the extent of hepatic *CD36* downregulation after bariatric surgery correlates with improvements in steatosis^53^. Acc1 is the rate-limiting step for de novo lipogenesis whereas Acc2 generates malonyl-CoA to allosterically inhibit Cpt1^54^. Liver-specific knockout of *Acc1* in mice protects against hepatic steatosis^55,56^, and pharmacological inhibition of *Acc* reduces de novo lipogenesis, enhances fatty acid oxidation, and reduces hepatic TG in preclinical and human studies^57^. Aco catalyzes β-oxidation of very long chain fatty acids, and mice lacking *Aco* develop severe microvesicular steatohepatitis^58,59^. Cpt1 is a mitochondrial enzyme that transfers fatty acids from the cytosol prior to β-oxidation, with *Cpt1a* being the hepatic isoform. *CPT1* expression is reduced in humans by 50% in NAFLD compared to normal subjects^60^. Therefore, increased expression of *Aco* and *Cpt1a* in *pNAPE-EcN* treated mice is consistent with protection against hepatosteatosis.

Treatment with *pNAPE-EcN* not only altered expression of fat metabolizing genes, but reduced expression of genes associated with inflammation including *Tnfα*, *Ccl2*, and *F4/80*. These changes are consistent with previous reports of anti-inflammatory effects of NAEs. For instance, C16:0NAE (*N*-palmitoylethanolamide) inhibits a wide variety of inflammatory activities including mast cell activation^25,26^; pattern recognition molecule induction of eicosanoids and cytokines and leukocyte infiltration^27–31^; ischemia-reperfusion injury^32^; dextran sodium sulfate induced colitis and enteric glial cell activation^61^; and diabetic retinopathy^33^. Both C18:1NAE and C18:0NAE have also been reported to exhibit anti-inflammatory effects^34,35^. In addition to the expression of inflammatory genes, initiation of the fibrotic response is another important step in the development of steatohepatitis. Although fibrosis was not visually observable in vehicle-treated *Ldlr*^−/−^ mice after 12 weeks of the Western diet, *Sma* and *Timp1* are both important molecular indicators that the fibrotic response has been initiated^46–48^, and their expression was elevated. Treatment with *pNAPE-EcN* suppressed their expression, suggesting that NAEs can also block the initiation of fibrosis. Together, our findings suggest that treatment with *pNAPE-EcN* has the potential to retard development of NASH in humans exposed to obesogenic diets.

In regards to cardiovascular disease, the most important findings of our study were that *pNAPE-EcN* reduced plasma cholesterol levels and necrosis in atherosclerotic lesions in the *Ldlr*^−/−^ mice. Our finding of reduced cholesterol levels concords with a previous report that administration of C18:1NAE (10 mg/kg/day i.p.) for 8 weeks to *ApoE*^−/−^ mice fed a Western diet lowered total and LDL cholesterol levels compared to vehicle treated mice^37^. Although a previous study with C18:1NAE in *ApoE*^−/−^ mice found reduced atherosclerotic lesion area^37^, we did not find that treatment with *pNAPE-EcN* significantly decreased atherosclerotic lesion size, but rather reduced necrotic area within the atherosclerotic lesion, consistent with an effect on reducing macrophage cell death. The contribution of macrophages to plaque morphology is size-independent and vulnerable plaques are characterized by the formation of the necrotic core and thinning of the fibrous cap. Our finding is particularly significant because lesion area is considered a poor predictor of clinical disease in humans^62^, with more advanced plaque features such as the necrotic core formation considered more important indicators^63^.

The anti-atherosclerotic effects of *pNAPE-EcN* treatment are consistent with the ability of NAEs to act as agonists of both PPARα and GPR119. PPARα agonists induce expression of ApoAI^21^ and decrease ApoC-III production^64^. ApoAI is the major protein component of HDL and facilitates efflux of cholesterol from macrophages via ABCA1, while ApoC-III is primarily a component of VLDL that increases plasma triglycerides by inhibiting lipoprotein lipase. *In vivo*, PPARα agonists such as fibrates increase HDL levels while modestly reducing LDL cholesterol levels^22^. In addition to driving cholesterol efflux, ApoAI may protect macrophages against necrosis, as ectopic expression of ApoAI in macrophages that are subsequently transplanted into Ldlr^−/−^; ApoAI^−/−^ mice leads to reduced necrotic core area without affecting aortic macrophage levels^65^. Agonism of GPR119 by NAEs may synergize with their effects on PPARα, as GPR119 activation was recently shown to increase ABCA1 expression in macrophages, and to thereby increase cholesterol efflux from macrophages^24^. Thus, agonism of both PPARα and GPR119 may mediate the anti-atherosclerotic effects of *pNAPE-EcN* treatment. Mechanisms underlying the effect of NA(P)Es on advanced plaque features warrant further investigation. Nevertheless, this protection against necrosis suggest the potential value of *pNAPE-EcN* as an adjuvant therapy for treating atherosclerosis, since ruptures of vulnerable plaques lead to occlusive thrombosis, myocardial infarction, and death in humans with cardiovascular disease.

Finally, we note that our strategy of using gut bacteria engineered to produce NAPEs offers advantages over two obvious alternative strategies. While altering the gut microbial composition through use of traditional pre- or pro-biotics may be useful^66,67^, our strategy offers the advantage of sustained delivery of precisely characterized therapeutic compounds using a bacterial strain that reliably colonizes. Alternatively, traditional oral delivery of purified NA(P)Es can also be employed, but we previously found that bacterial biosynthesis of NAPE in the intestinal tract was more potent and efficacious than oral administration of purified NAPE^19^, and that colonization of *pNAPE-EcN* provided protection even weeks after ending administration^12^. For these reasons, incorporating *pNAPE-EcN* or other bacteria engineered to express NAPE into the gut microbiota appears to have significant potential as a long-term adjuvant strategy for slowing the development of metabolic syndrome and cardiometabolic disease.

## Methods

### Bacterial strains and preparation

*EcN* (Ardey Pharm, GmbH) was transformed with *pNAPE* (*At1g78690*) as previously described^12^. Briefly, for expression of *pNAPE* in *EcN*, *pQE-80L* (QIAGEN) was modified by removing one lac operator to enable a basal expression of inserted genes without isopropyl β-D-1-thiogalactopyranoside induction. The *At1g78690* gene was obtained by high-fidelity PCR using pDEST-At1g78690^68^ as a template, and subcloned in frame into *pQE-80L1* digested with BamHI and SacI.

### Animal studies

Public Health Services guidelines regarding the use and care of laboratory animals were observed. All procedures involving animals were approved by the Vanderbilt University Institutional Animal Care and Use Committee. Forty male *Ldlr*^−/−^ mice on a C57BL/6J background were purchased at 5 weeks old (Jackson Laboratories, Bar Harbor, ME; stock number 002207) and housed in standard mouse cages with bedding at the Vanderbilt University animal facility in a 12-hour light/12-hour dark cycle, with food and water given *ad libitum*. The animals were acclimated for 1 week and maintained on standard rodent chow (LabDiet 5001). The animals were divided into 4 experimental groups of 10 mice each, and housed individually (1 mouse per cage) to measure food intake. To reduce the gut microbiota prior to bacteria administration, the animals were gavaged with an antibiotic cocktail (10 ul/g body weight) containing amphotericin B (0.3 mg/ml), metronidazole (10 mg/ml), vancomycin (0.5 mg/ml), neomycin (10 mg/ml), and ampicillin (10 mg/ml) for 3 consecutive days. Three experimental groups were switched to a Western diet (Harlan Laboratories, catalog #TD.10885) containing 45% fat by kcal, while the fourth group was switched to a low fat control diet (LFD) (Harlan Laboratories, Inc, catalog #TD.120724). Groups fed the Western diet (WD) received standard drinking water containing vehicle (0.125% gelatin) as a control, control *Nissle 1917* (*pEcN)* (5 × 10^9^ CFU/ml suspended in 0.125% gelatin), or NAPE-expressing *Nissle 1917 (pNAPE-EcN)* (5 × 10^9^ CFU/ml suspended in 0.125% gelatin). The chow-fed group received standard drinking water containing vehicle as an additional control group. Fresh drinking water and bacteria preparations were given 3 times a week. The treatments continued for 12 weeks before euthanasia. During this period, body weights and food intake were measured twice weekly. Body composition was measured by nuclear magnetic resonance analyses (Bruker Minispec MQ10). Before the morning of euthanasia, animals were fasted overnight. Under isofluorane anesthesia, blood samples were collected via retro-orbital venous plexus puncture. The animals were then euthanized via isofluorane overdose followed by cervical dislocation. The left ventricle of the heart was flushed with 30 mL saline. Liver and adipose samples were collected as tissues frozen in liquid nitrogen and as samples fixed in neutral buffered formalin for sectioning. The entire aorta was dissected for *en face* preparation as previously described^45^. The heart with the proximal aorta was embedded in optimal cutting temperature compound and frozen on dry ice. Preparation and analyses of tissues are described below.

### Hepatic TG and NAPE measurements

For hepatic TG measurements, lipids were extracted using chloroform/methanol (2:1), and individual lipid classes were separated by thin-layer chromatography. The TGs were scraped from the plate, and the fatty acids methylated using BF3/methanol. Fatty acid methyl esters were analyzed using an Agilent 7890 A gas chromatograph equipped with flame ionization detectors and a capillary column (SP2380, 0.25 mm × 30 m, 0.25 μm film; Supelco). The esters were identified by comparing the retention times to those of known standards. Trieicosenoin (C20:1) was added to the total lipid extract, serving as an internal standard and permitting quantitation of the amount of TGs in the sample.

### Liver staining and immunohistochemistry

For staining and immunohistochemistry procedures, frozen liver cyrosections were prepared at 10 microns thick on microscope slides. For Oil Red O staining, freshly cut liver sections were fixed in neutral buffered formalin before rinsing in water and equilibration in 60% isopropanol. Slides were then stained with Oil Red O (Sigma, Aldrich, St. Louis, MO) working solution prepared in 100% isopropanol. For H&E staining of liver sections, slides were stained with hematoxylin (Richard-Allan), Bluing Reagent (Richard-Allan), and Eosin-Y (Richard-Allan). For Trichrome Blue staining, slides were stained with a Masson’s Trichrome stain kit (Dako, North America, Inc). For *F4/80* immunohistochemistry, enzymatic induced antigen retrieval was performed using Proteinase K (Dako, North America, Inc) for 5 minutes. Slides were incubated with anti-*F4/80* (NB600-404, Novus Biologicals) for at a 1:1000 dilution and then incubated in a rabbit anti-rat secondary (BA-4001, Vector Laboratories, Inc.) at a 1:200 dilution. The Bond Polymer Refine detection system was used for visualization. After staining, slides were dehydrated, cleared and coverslipped. Immunostained tissue microarray slides were imaged on a Leica SCN400 Slide Scanner (Leica Biosystems). Tissue cores were imaged at 20X magnification to a resolution of 0.5 μm/pixel. Cells were identified utilizing standard Ariol analysis scripts. (Leica) Upper and lower thresholds for color, saturation, intensity, size were set for both blue Hematoxylin staining of nuclei and for brown DAB reaction products, so that the brown (DAB) positive cells can be distinguished from blue (Hematoxylin only) negative cells. The area of positive F4/80 staining area was calculated as a percent of the total analyzed area.ddc‘1×.

### Hepatic gene expression

Total RNA was extracted from frozen liver tissue using the RNeasy Micro Kit (QIAGEN), and transcribed into cDNA using a cDNA reverse transcription kit (Applied Biosystems). Gene expression levels were quantified by RT-PCR using SYBR Green PCR Master Mix (QIAGEN) and the 7500 Real Time PCR system (Applied Biosystems). Primer sequences are described in Supplementary Table 1. Relative quantification of gene expression with real-time PCR data was calculated relative to *Ppia* (*cyclophilin*).

### Quantification of Arterial Lesions

Cryosections of the proximal aorta that were 10 microns thick were prepared starting at the aortic sinus and continuing 300 microns distally on microscope slides, according to the method of Paigen *et al*.^69^ and adapted for computer analysis^70^. Sections were stained with Oil Red O and counterstained with hematoxylin. Additional slides were stained with H&E and Trichrome as described above. En face aortas were stained with Sudan IV solution (Sigma-Aldrich). The images of the aorta were captured and analyzed with an imaging system (KS 300 Release 2.0; Kontron Electronik GmbH).

### Serum Cholesterol

Serum concentrations of total cholesterol were determined by enzymatic methods (Raichem) according to the manufacturer’s instructions. Absorbance was measured at 500 nm.

### Statistical Analysis

Statistical analysis was performed using GraphPad Prism 7. A *P* value of less than 0.05 was considered statistically significant. For 2-way repeated-measures ANOVA, when overall significance for treatment effect was found, the Bonferroni’s multiple comparison post-hoc test was used to determine differences between treatment groups. For 1-way ANOVA, when overall significance for treatment effect was found, the Dunnett’s multiple comparison post-hoc test was used to determine groups that differed from the control group.

## Supplementary information

Supplementary Data

## Data Availability

The datasets generated during the current study are available from the corresponding author on reasonable request.
