# Supplementary material for: Administration of N-Acyl-Phosphatidylethanolamine Expressing Bacteria to Low Density Lipoprotein Receptor−/− Mice Improves Indices of Cardiometabolic Disease
Source: Sci Rep. 2019 Jan 23;9:420. doi: 10.1038/s41598-018-37373-1 (PMC6344515; doi:10.1038/s41598-018-37373-1)
Supplement: Supplementary file 1 — Supplementary Data [file 41598_2018_37373_MOESM1_ESM.docx]

**Supplementary Material**

Administration of N-Acyl-Phosphatidylethanolamine Expressing Bacteria to Low Density Lipoprotein Receptor-/- Mice Improves Indices of Cardiometabolic Disease.

Linda S. May-Zhang^1^, Zhongyi Chen^1^, Noura S. Dosoky^1^, Patricia G. Yancey^2^, Kelli L. Boyd^3^, Alyssa H. Hasty^4^, MacRae F. Linton^2^, Sean S. Davies^1^

^1^556 Robinson Research Building, Division of Clinical Pharmacology, Department of Pharmacology, 2220 Pierce Avenue, Vanderbilt University, Nashville, TN 37221, USA

^2^312 Preston Research Building, Department of Medicine, Division of Cardiovascular Medicine, Vanderbilt Medical Center, 2220 Pierce Avenue, Nashville, TN 37232, USA

^3^AA-6206 Medical Center North, Department of Pathology, Microbiology, and Immunology, Vanderbilt Medical Center, 1211 Medical Center Drive, Nashville, TN 37232, USA

^4^813 Light Hall, Department of Molecular Physiology and Biophysics, Vanderbilt University, 2220 Pierce Avenue, Nashville, TN 37232, USA

| **Supplementary Figure 1.**  **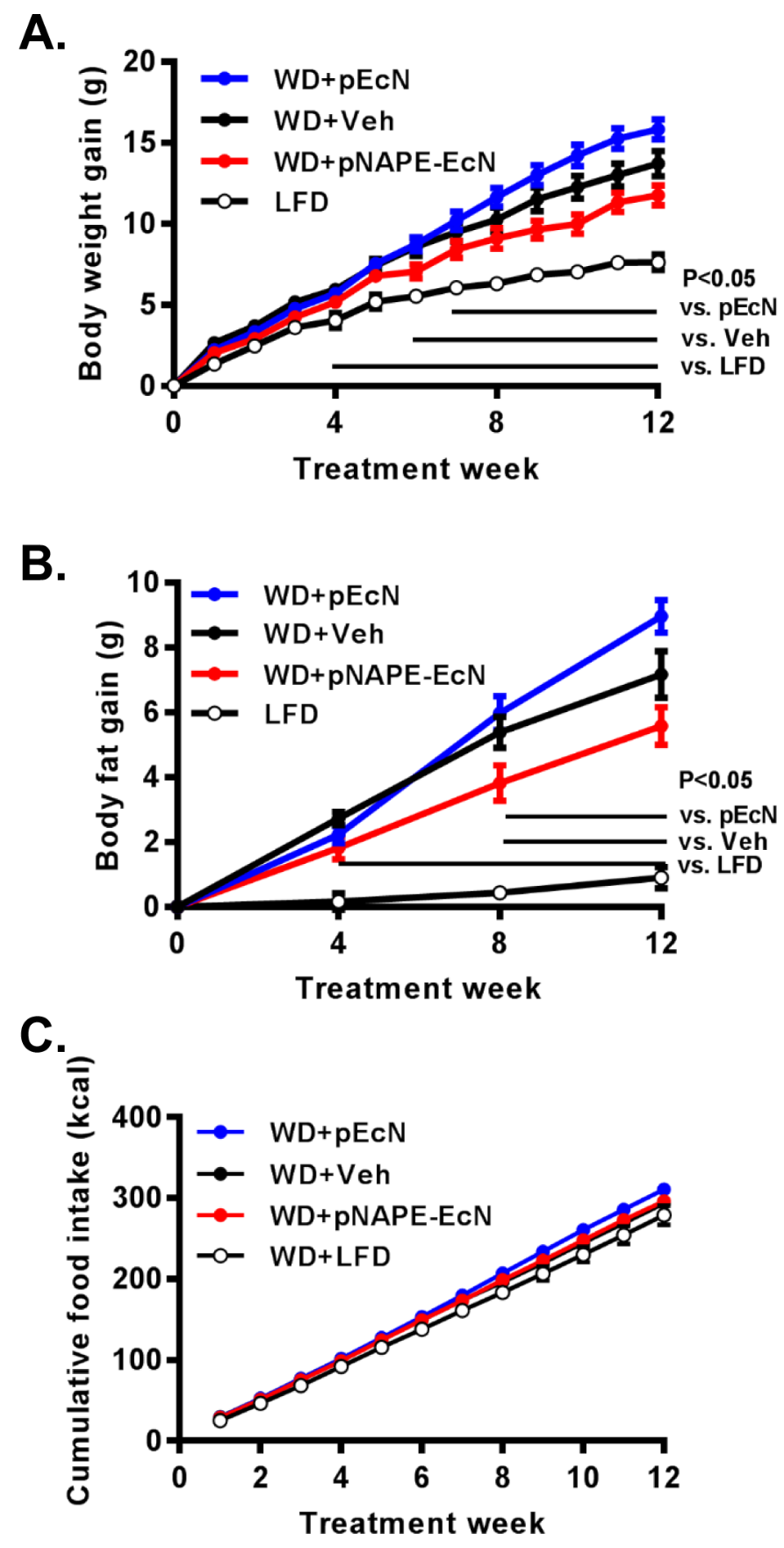**  **Supplementary Figure 1. *pNAPE-EcN*, but not *pEcN*, inhibits gain in body weight and fat mass.** All values are mean ± SEM (n=10 mice per group). pEcN, Veh, and pNAPE-EcN groups were fed WD for 12 weeks and compared to LFD as an additional control group. Solid bars indicate time points with significant differences (P<0.05) between *pNAPE-EcN* and other groups (2-way repeated measures ANOVA with Dunnett’s multiple comparison test). (a) Effect on gain of body weight from start of treatment. (b) Effect on gain of body fat.  **Supplementary Figure 2.**  **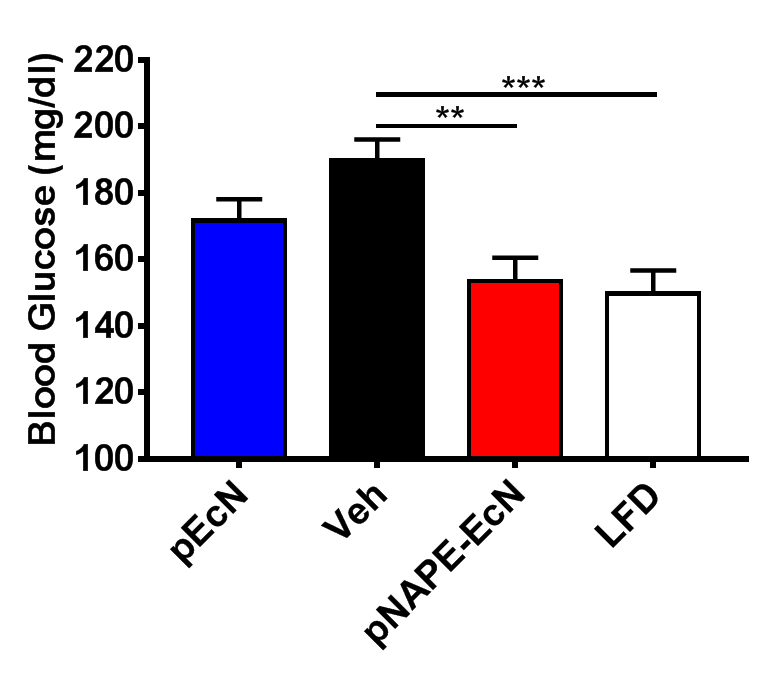**  **Supplementary Figure 2. *pNAPE-EcN* significantly reduces fasting blood glucose levels compared Veh treated group, to levels similar to LFD (n=10 animals per group) at 8 weeks.** Values are represented as mean ± SEM. Statistical significance is **P<0.01; ***P<0.005 by 1-way ANOVA with Dunnett’s multiple comparisons test.  **Supplementary Table 1.** | | |
| --- | --- | --- |
| *Gene name* | *Forward sequence* | *Reverse sequence* |
| *Acc1* | GCCTCTTCCTGACAGAG | TGACTGCCGAAACATCTCTG |
| *Acc2* | GCAGGTAAGTAAGTGTGCTG | GCCACCAGTTCCATTCTCAG |
| *Aco* | GTGCAGCTCAGAGTCTGTCCAA | TACTGCTGCGTCTGAAAATCCA |
| *Angplt3* | TCTACTGTGATACCCAATCAGGC | CATGTTTCGTTGAAGTCCTGTGA |
| *Angplt4* | CATCCTGGGACGAGATGAACT | TGACAAGCGTTACCACAGGC |
| *Ccl3* | TTCTCTGTACCATGACACTCTGC | TTCTCTGTACCATGACACTCTGC |
| *Ccr2* | TGTGATTGACAAGCACTTAGACC | TGGAGAGATACCTTCGGAACTT |
| *Ccl2* | ACTGAAGCCAGCTCTCTCTTCCTC | TTCCTTCTTGGGGTCAGCACAGAC |
| *Cd36* | CCTTAAAGGAATCCCCGTGT | TGCATTTGCCAATGTCTAGC |
| *Cd68* | CCATCCTTCACGATGACACCT | GGCAGGGTTATGAGTGACAGTT |
| *Cpt1* | GCTGGAGGTGGCTTTGGT | GCTTGGCGGATGTGGTTC |
| *Emr1* | TGCATCTAGCAATGGACAGC | GCCTCCTGGATCCATTTGAA |
| *Igam* | TCCGGTAGCATCAACAACAT | GGTGAAGTGAATCCGGAACT |
| *Il-6* | TAGTCCTTCCTACCCCAATTTCC | TTGGTCCTTAGCCACTCCTTC |
| *Il-10* | GCTCTTACTGACTGGCATGAG | CGCAGCTCTAGGAGCATGTG |
| *Lpl* | GGTTGCGCGTAGAGAGGATG | CTCACGCTCTGACATGCCTTC |
| *Pparα* | GTACGGTGTGTATGAAGCCATCTT | GCCGTACGCGATCAGCAT |
| *Pparδ* | GCCATATTCCCAGGCTGTC | CAGCACAAGGGTCATCTGTG |
| *Pparγ* | GCCCTTTGGTGACTTTATGGA | GCAGCAGGTTGTCTTGGATG |
| *Sma* | GCATCCACGAAACCACCTA | CACGAGTAACAAATCAAAGC |
| *Tgf-β* | CTCCCGTGGCTTCTAGTGC | GCCTTAGTTTGGACAGGATCTG |
| *Timp1* | GCAACTCGGACCTGGTCATAA | CGGCCCGTGATGAGAAACT |
| *Tnf* | CCATTCCCTGAGTTCTGCAAAG | GCAAATATAAATAGAGGGGGGC |
| *Ppia* | AGCACTGGAGAGAAAGGATTTGG | TCTTCTTGCTGGTCTTGCCATT |

**Supplementary Table 1.** Primer sequences used for RT-PCR analysis.

**Supplementary Table 2.**

| Hepatic gene expression (fold change compared to LFD control) | | | | |
| --- | --- | --- | --- | --- |
|  | **Veh** | **pEcN** | **pNAPE-EcN** | Significance (among HFD groups) |
| *Il-10* | 0.83±0.10 | 2.03±0.23 | 1.02±0.08 | P<0.001 pNAPE-EcN vs pEcN  P<0.001 pEcN vs veh |
| *Tgf-β* | 0.49±0.12 | 1.07±0.18 | 0.96±0.17 | N.S. |
| *Angplt3* | 1.04±0.10 | 0.98±0.10 | 0.84±0.10 | N.S |
| *Angplt4* | 0.12±0.03 | 0.18±0.06 | 0.12±0.03 | N.S. |
| *Lpl* | 0.54±0.08 | 0.87±0.09 | 0.81±0.16 | N.S. |
| *Il-6* | 0.83±0.10 | 2.03±0.23 | 1.76±0.58 | P<0.05 pEcN vs Veh |
| *Ppar-α* | 0.96±0.08 | 0.76±0.10 | 0.81±0.11 | N.S |
| *Ppar-δ* | 0.11±0.01 | 0.10±0.01 | 0.09±0.02 | N.S. |
| *Ppar-γ* | 4.82±0.88 | 5.14±0.88 | 4.02±0.53 | N.S. |
| *Cd68* | 1.27±0.13 | 1.76±0.22 | 0.99±0.09 | N.S. |

**Supplementary Table 2.** Effect of treatments on hepatic mRNA expression of *additional hepatic genes related to inflammation and metabolism.* Values are represented as mean ± SEM. Statistical significance is *P<0.05; **P<0.01; ****P<0.001 by 1-way ANOVA with Dunnett’s multiple comparisons test.
